# 3-[(*E*)-2-(5,7-Dichloro-8-hydroxy­quinolin-2-yl)vin­yl]-4-hydroxy­phenyl acetate

**DOI:** 10.1107/S1600536809001950

**Published:** 2009-02-11

**Authors:** Łukasz Ponikiewski, Jacek E. Nycz

**Affiliations:** a Department of Inorganic Chemistry, Faculty of Chemistry, Gdańsk University of Technology, 11/12 G. Narutowicz St., 80952 Gdańsk, Poland; b Department of Organic Chemistry, University of Silesia, 9 Szkolna St., 40007 Katowice, Poland

## Abstract

The two symmetry independent mol­ecules of the title compound, C_19_H_13_Cl_2_NO_4_, show similar conformations with the acetyl group twisted strongly relative to the remaining, virtually flat (r.m.s. deviations = 0.0173 and 0.0065 Å), part of the mol­ecule. The hydroxyl groups of the 8-hydroxy­quinoline residues are involved in intra­molecular O—H⋯N hydrogen bonds, which, in one case, forms a part of a three-center inter­action. Inter­molecular O—H⋯O hydrogen bonds assemble the mol­ecules into a one-dimensional polymeric structure extended along the *a* axis. The 4-hydroxy­phenyl group of one mol­ecule forms an O—H⋯O hydrogen bond, in which the hydroxyl H atom is disordered, with its inversion center counterpart.

## Related literature

For the biological activity and applications of chloro­quino­lines, see: O’Neill *et al.* (1998 ▶); Blauer *et al.* (1998 ▶); Egan *et al.* (2000 ▶); Zouhiri *et al.* (2000 ▶). For the structure of a similar compound, see: Chojnacki *et al.* (2007 ▶).
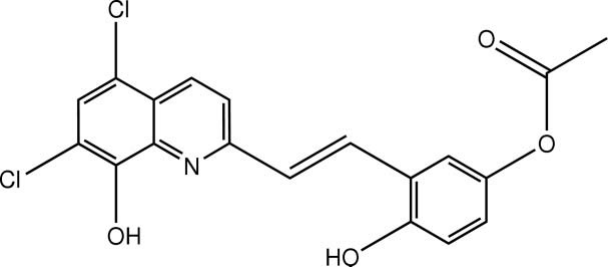


## Experimental

### 

#### Crystal data

C_19_H_13_Cl_2_NO_4_
*M* *_r_* = 390.20
Triclinic, 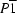

*a* = 7.3274 (15) Å
*b* = 10.449 (2) Å
*c* = 21.550 (4) Å
α = 84.89 (3)°
β = 89.50 (3)°
γ = 87.31 (3)°
*V* = 1641.6 (6) Å^3^
*Z* = 4
Mo *K*α radiation
μ = 0.42 mm^−1^
*T* = 120 (2) K
0.36 × 0.29 × 0.11 mm

#### Data collection

Oxford Diffraction KM4CCD κ-geometry diffractometer
Absorption correction: none
13404 measured reflections
7118 independent reflections
5533 reflections with *I* > 2σ(*I*)
*R*_int_ = 0.024

#### Refinement

*R*[*F* ^2^ > 2σ(*F* ^2^)] = 0.043
*wR*(*F* ^2^) = 0.109
*S* = 1.06
7118 reflections
491 parameters
4 restraints
H atoms treated by a mixture of independent and constrained refinement
Δρ_max_ = 0.54 e Å^−3^
Δρ_min_ = −0.24 e Å^−3^

### 

Data collection: *CrysAlis CCD* (Oxford Diffraction, 2006 ▶); cell refinement: *CrysAlis RED* (Oxford Diffraction, 2006 ▶); data reduction: *CrysAlis RED*; program(s) used to solve structure: *SHELXS97* (Sheldrick, 2008 ▶); program(s) used to refine structure: *SHELXL97* (Sheldrick, 2008 ▶); molecular graphics: *ORTEP-3 for Windows* (Farrugia, 1997 ▶); software used to prepare material for publication: *WinGX* publication routines (Farrugia, 1999 ▶).

## Supplementary Material

Crystal structure: contains datablocks I, global. DOI: 10.1107/S1600536809001950/gk2178sup1.cif

Structure factors: contains datablocks I. DOI: 10.1107/S1600536809001950/gk2178Isup2.hkl

Additional supplementary materials:  crystallographic information; 3D view; checkCIF report

## Figures and Tables

**Table 1:** Hydrogen-bond geometry (Å, °)

*D*—H⋯*A*	*D*—H	H⋯*A*	*D*⋯*A*	*D*—H⋯*A*
O1—H1⋯N1	0.78 (3)	2.14 (3)	2.667 (2)	125 (3)
O2—H2⋯O8	0.88 (3)	1.84 (3)	2.675 (2)	159 (3)
O5—H5⋯N2	0.77 (3)	2.20 (3)	2.692 (2)	122 (3)
O5—H5⋯O4^i^	0.77 (3)	2.38 (3)	2.990 (2)	138 (3)
O6—H6*A*⋯O6^ii^	0.829 (19)	2.12 (3)	2.892 (3)	156 (5)
O6—H6*B*⋯O4^i^	0.844 (10)	2.445 (11)	3.130 (3)	138.9 (18)

Symmetry codes: (i) 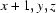; (ii) 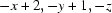.

**Fig. 1.**
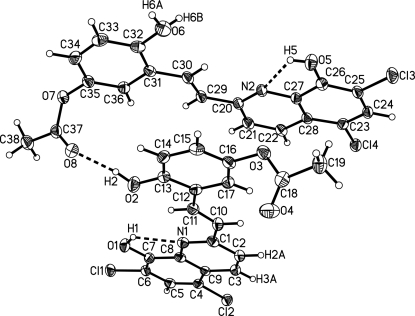
Structure of 3-[(E)-2-(5,7-dichloro-8-hydroxyquinolin-2-yl)vinyl]-4-hydroxyphenyl acetate showing the atomic numbering scheme. Displacement ellipsoids are drawn at the 50% probability level.

**Fig. 2.**
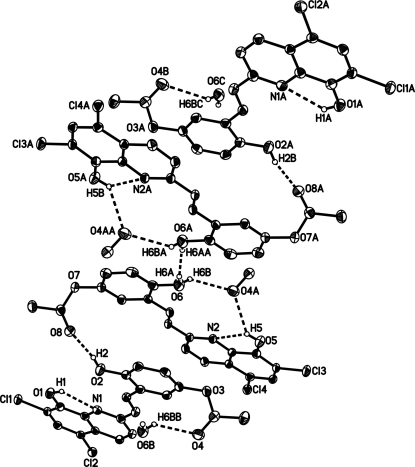
Hydrogen bonds between 3-[(E)-2-(5,7-dichloro-8-hydroxyquinolin-2-yl)vinyl]-4-hydroxyphenyl acetate molecules. For clarity, all H atoms, except O—H, have been omitted. Symmetry code for the atoms designated with A: 2 - x,1 - y,-z.

**Fig. 3.**
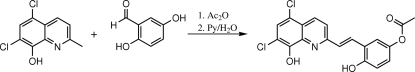
Preparation of the title compound.

## supplementary crystallographic information

### Comment

5,7-Dichloro-2-methylquinolin-8-ol (chlorquinaldol) is a well known antiseptic and generally chloroquinolines are a class of compounds of great interest from a synthetic, theoretical and practical point of view (O'Neill *et al., *1998; Blauer *et al., *1998; Egan *et al., *2000). Styryl derivatives of chloroquinolines have recently attracted special attention due to their antiviral activity (Zouhiri *et al., *2000).

The title compound was synthesized in the reaction of 2,5-dihydroxybenzaldehyde with an equimolar quantity of 5,7-dichloro-2-methylquinolin-8-ol in Ac_2_O followed by partial hydrolysis in py/H_2_O system (Fig. 3).

The molecular structure of title compound is presented in Fig.1. Both molecules from the asymmetric unit exist in an *E* configuration with respect to the ethenyl C=C bond [C10δb C11 = 1.328 (3)Å and C29δb C30 - 1.330 (3) Å]. The dihedral angles between the phenyl and quinoline rings are 5.22 (8)° and 9.80 (8)°.

The crystal structure is stabilized by intramolecular and intermolecular hydrogen bonding. Intramolecular O—H···N contacts result in the formation of planar five-membered rings (O1/H1/N1/C8/C7 and O5/H5/N2/C27/C26). The intermolecular interactions O2—H2···O8 and O5—H5···O4^i ^(symmetry codes: (i) *x* + 1, *y*, *z*) assemble molecules into one-dimensional polymeric structure extended along the *a* axis. These assemblies are further joined *via* O6—H6A···O6A^ii^ [symmetry code: (ii) -*x* + 2, -*y* + 1, -*z*] hydrogen bonds in which the H atoms are disordered and have 0.5 occupancy (Fig. 2.) An alternative position of the H6A could not be located from a difference Fourier map.

### Experimental

To the solution of 5,7-dichloro-2-methylquinolin-8-ol (1.14 g, 5 mmol) in Ac_2_O (25 ml) at 303 K, 2,5-dihydroxybenzaldehyde (0.69 g, 5 mmol) was added. The reaction mixture was stirred at 373 K for 16 h. Next, the solvent was evaporated and the py/H_2_O system (4:1, 20 ml) was added and the reaction mixture was stirred at 373 K for 3 h. The solvent was evaporated and residue was purified by crystallization using EtOH, THF and finally CH_3_CN.

### Refinement

Carbon-bound H atoms were included in idealized positions and refined as riding atoms with aromatic and methylene C—H = 0.95 Å, methyl C—H = 0.98Å and *U*_iso_(H) = 1.2 U*_e.g_*(C) for aromatic and methylene C—H and *U*_iso_(H) = 1.5 *U*_eq_(C) for methyl group. The OH group H atoms H1, H2, H5 were located in a difference Fourier map and fully refined. The O6—H group is disordered. In one site (50% occupancy) it is involved in strong O6—H6A···O6(-*x* + 2, -*y* + 1, -*z*) hydrogen bonding. H6A was located in a difference Fourier map and in the refinement process the O6—H6A distance was restrained to 0.82 (2) Å and isotropic displacement parameter refined. An alternative position of this H atom (H6B) could not be determined from difference Fourier maps. Position of this H atom was calculated assuming its H-bond interaction with O4 and restrained intramolecular distances O6—H6B 0.85 (1), H30A···H6B 2.20 (1) and C32···H6B 1.83 (1) Å. Isotropic displacement parameter of H6A was refined.

### Crystal data

**Table:**

C_19_H_13_Cl_2_NO_4_	*Z* = 4
*M**_r_* = 390.20	*F*(000) = 800
Triclinic, *P*1	*D*_x_ = 1.579 Mg m^−^^3^
Hall symbol: -P 1	Mo *K*α radiation, λ = 0.71073 Å
*a* = 7.3274 (15) Å	Cell parameters from 8303 reflections
*b* = 10.449 (2) Å	θ = 2.0–32.3°
*c* = 21.550 (4) Å	µ = 0.42 mm^−^^1^
α = 84.89 (3)°	*T* = 120 K
β = 89.50 (3)°	Block, orange
γ = 87.31 (3)°	0.36 × 0.29 × 0.11 mm
*V* = 1641.6 (6) Å^3^	

### Data collection

**Table:**

Oxford Diffraction KM4CCD κ-geometry diffractometer	5533 reflections with *I* > 2σ(*I*)
Radiation source: fine-focus sealed tube	*R*_int_ = 0.024
graphite	θ_max_ = 27.0°, θ_min_ = 4.1°
Detector resolution: 8.1883 pixels mm^-1^	*h* = −8→9
ω scans, 0.75 deg width	*k* = −13→10
13404 measured reflections	*l* = −27→27
7118 independent reflections	

### Refinement

**Table:**

Refinement on *F*^2^	Primary atom site location: structure-invariant direct methods
Least-squares matrix: full	Secondary atom site location: difference Fourier map
*R*[*F*^2^ > 2σ(*F*^2^)] = 0.043	Hydrogen site location: inferred from neighbouring sites
*wR*(*F*^2^) = 0.109	H atoms treated by a mixture of independent and constrained refinement
*S* = 1.06	*w* = 1/[σ^2^(*F*_o_^2^) + (0.0649*P*)^2^] where *P* = (*F*_o_^2^ + 2*F*_c_^2^)/3
7118 reflections	(Δ/σ)_max_ = 0.001
491 parameters	Δρ_max_ = 0.54 e Å^−^^3^
4 restraints	Δρ_min_ = −0.24 e Å^−^^3^

### Special details

**Table:**

Geometry. All e.s.d.'s (except the e.s.d. in the dihedral angle between two l.s. planes) are estimated using the full covariance matrix. The cell e.s.d.'s are taken into account individually in the estimation of e.s.d.'s in distances, angles and torsion angles; correlations between e.s.d.'s in cell parameters are only used when they are defined by crystal symmetry. An approximate (isotropic) treatment of cell e.s.d.'s is used for estimating e.s.d.'s involving l.s. planes.
Refinement. Refinement of *F*^2^ against ALL reflections. The weighted *R*-factor *wR* and goodness of fit *S* are based on *F*^2^, conventional *R*-factors *R* are based on *F*, with *F* set to zero for negative *F*^2^. The threshold expression of *F*^2^ > σ(*F*^2^) is used only for calculating *R*-factors(gt) *etc*. and is not relevant to the choice of reflections for refinement. *R*-factors based on *F*^2^ are statistically about twice as large as those based on *F*, and *R*- factors based on ALL data will be even larger.

### Fractional atomic coordinates and isotropic or equivalent isotropic displacement parameters (Å^2^)

**Table:**

	*x*	*y*	*z*	*U*_iso_*/*U*_eq_	Occ. (<1)
Cl1	−0.05058 (7)	−0.48122 (4)	0.39447 (2)	0.02868 (13)	
Cl2	−0.32326 (7)	−0.14842 (5)	0.55244 (2)	0.02702 (13)	
Cl3	0.51139 (7)	1.02105 (4)	0.29549 (2)	0.02920 (13)	
Cl4	0.34086 (7)	0.64476 (4)	0.46981 (2)	0.02556 (12)	
N1	−0.0213 (2)	−0.00314 (15)	0.34122 (7)	0.0220 (3)	
N2	0.6083 (2)	0.55562 (14)	0.25358 (7)	0.0206 (3)	
O1	0.0315 (2)	−0.24598 (15)	0.31376 (7)	0.0275 (3)	
H1	0.059 (4)	−0.177 (3)	0.3008 (13)	0.049 (9)*	
O2	0.2381 (2)	0.12385 (13)	0.14624 (7)	0.0324 (4)	
H2	0.306 (4)	0.101 (3)	0.1151 (13)	0.051 (8)*	
O3	0.22826 (19)	0.64374 (13)	0.17441 (7)	0.0272 (3)	
O4	−0.0706 (2)	0.66859 (14)	0.15324 (8)	0.0415 (4)	
O5	0.6377 (2)	0.80890 (14)	0.22189 (7)	0.0280 (3)	
H5	0.671 (4)	0.746 (3)	0.2085 (13)	0.054 (9)*	
O6	0.8761 (2)	0.47684 (14)	0.05196 (8)	0.0320 (3)	
H6B	0.936 (2)	0.4981 (17)	0.0825 (5)	0.11 (3)*	0.50
H6A	0.918 (7)	0.495 (5)	0.0166 (13)	0.057 (18)*	0.50
O7	0.74929 (18)	−0.04271 (12)	0.07725 (6)	0.0263 (3)	
O8	0.44540 (19)	−0.00398 (14)	0.06726 (7)	0.0315 (3)	
C1	−0.0496 (3)	0.11986 (18)	0.35315 (9)	0.0229 (4)	
C2	−0.1381 (3)	0.15372 (18)	0.40825 (9)	0.0245 (4)	
H2A	−0.1588	0.2419	0.4150	0.029*	
C3	−0.1942 (3)	0.06119 (18)	0.45183 (9)	0.0229 (4)	
H3A	−0.2528	0.0846	0.4889	0.027*	
C4	−0.2139 (2)	−0.17651 (18)	0.48241 (9)	0.0206 (4)	
C5	−0.1781 (2)	−0.30041 (18)	0.46866 (9)	0.0220 (4)	
H5A	−0.2092	−0.3702	0.4975	0.026*	
C6	−0.0950 (3)	−0.32370 (18)	0.41148 (9)	0.0226 (4)	
C7	−0.0468 (3)	−0.22522 (18)	0.36942 (9)	0.0220 (4)	
C8	−0.0785 (2)	−0.09591 (18)	0.38460 (9)	0.0211 (4)	
C9	−0.1646 (2)	−0.07036 (17)	0.44149 (9)	0.0193 (4)	
C10	0.0138 (3)	0.22128 (18)	0.30779 (9)	0.0240 (4)	
H10A	−0.0067	0.3081	0.3169	0.029*	
C11	0.0983 (3)	0.19915 (18)	0.25467 (9)	0.0233 (4)	
H11A	0.1183	0.1116	0.2467	0.028*	
C12	0.1638 (2)	0.29527 (18)	0.20718 (9)	0.0216 (4)	
C13	0.2360 (3)	0.25260 (18)	0.15143 (9)	0.0235 (4)	
C14	0.2986 (3)	0.33822 (19)	0.10404 (9)	0.0244 (4)	
H14A	0.3448	0.3075	0.0665	0.029*	
C15	0.2936 (3)	0.46913 (19)	0.11148 (9)	0.0250 (4)	
H15A	0.3372	0.5287	0.0795	0.030*	
C16	0.2238 (3)	0.51087 (18)	0.16657 (9)	0.0233 (4)	
C17	0.1590 (3)	0.42768 (18)	0.21369 (9)	0.0235 (4)	
H17A	0.1109	0.4598	0.2506	0.028*	
C18	0.0700 (3)	0.71436 (18)	0.16649 (9)	0.0260 (4)	
C19	0.0972 (3)	0.85296 (19)	0.17313 (10)	0.0330 (5)	
H19A	−0.0143	0.8924	0.1900	0.050*	
H19B	0.1246	0.8969	0.1322	0.050*	
H19C	0.1992	0.8607	0.2015	0.050*	
C20	0.5942 (2)	0.42938 (17)	0.26808 (9)	0.0207 (4)	
C21	0.5229 (3)	0.38022 (18)	0.32605 (9)	0.0238 (4)	
H21A	0.5151	0.2900	0.3351	0.029*	
C22	0.4651 (3)	0.46163 (17)	0.36918 (9)	0.0231 (4)	
H22A	0.4176	0.4284	0.4082	0.028*	
C23	0.4206 (3)	0.69136 (18)	0.39503 (9)	0.0214 (4)	
C24	0.4301 (2)	0.81905 (18)	0.37680 (9)	0.0218 (4)	
H24A	0.3893	0.8814	0.4039	0.026*	
C25	0.5008 (3)	0.85796 (17)	0.31761 (9)	0.0217 (4)	
C26	0.5643 (3)	0.77065 (18)	0.27752 (9)	0.0216 (4)	
C27	0.5491 (2)	0.63696 (17)	0.29608 (9)	0.0201 (4)	
C28	0.4767 (2)	0.59576 (17)	0.35519 (9)	0.0200 (4)	
C29	0.6498 (3)	0.34132 (17)	0.22142 (9)	0.0218 (4)	
H29A	0.6386	0.2518	0.2319	0.026*	
C30	0.7150 (3)	0.37867 (17)	0.16535 (9)	0.0218 (4)	
H30A	0.7306	0.4682	0.1563	0.026*	
C31	0.7652 (2)	0.29585 (17)	0.11613 (9)	0.0203 (4)	
C32	0.8451 (3)	0.34769 (18)	0.06051 (9)	0.0236 (4)	
C33	0.8925 (3)	0.2713 (2)	0.01282 (9)	0.0280 (4)	
H33A	0.9492	0.3082	−0.0239	0.034*	
C34	0.8578 (3)	0.1428 (2)	0.01846 (9)	0.0273 (4)	
H34A	0.8884	0.0905	−0.0143	0.033*	
C35	0.7775 (3)	0.09090 (18)	0.07278 (9)	0.0228 (4)	
C36	0.7329 (3)	0.16355 (17)	0.12123 (9)	0.0214 (4)	
H36A	0.6802	0.1245	0.1582	0.026*	
C37	0.5759 (3)	−0.07926 (19)	0.07142 (9)	0.0255 (4)	
C38	0.5673 (3)	−0.22131 (19)	0.06933 (10)	0.0319 (5)	
H38A	0.4973	−0.2393	0.0328	0.048*	
H38B	0.5076	−0.2582	0.1072	0.048*	
H38C	0.6914	−0.2597	0.0666	0.048*	

### Atomic displacement parameters (Å^2^)

**Table:**

	*U*^11^	*U*^22^	*U*^33^	*U*^12^	*U*^13^	*U*^23^
Cl1	0.0327 (3)	0.0194 (2)	0.0345 (3)	0.00009 (19)	−0.0010 (2)	−0.0062 (2)
Cl2	0.0297 (3)	0.0280 (3)	0.0239 (2)	−0.00402 (19)	0.0061 (2)	−0.00418 (19)
Cl3	0.0367 (3)	0.0158 (2)	0.0351 (3)	0.00011 (19)	0.0011 (2)	−0.00306 (19)
Cl4	0.0294 (3)	0.0246 (2)	0.0233 (2)	−0.00214 (19)	0.00264 (19)	−0.00533 (19)
N1	0.0209 (8)	0.0239 (8)	0.0211 (8)	−0.0022 (6)	−0.0006 (6)	−0.0014 (7)
N2	0.0215 (8)	0.0182 (8)	0.0226 (8)	−0.0004 (6)	−0.0022 (6)	−0.0050 (6)
O1	0.0350 (8)	0.0255 (8)	0.0227 (7)	−0.0010 (6)	0.0048 (6)	−0.0061 (6)
O2	0.0409 (9)	0.0216 (7)	0.0352 (9)	−0.0023 (6)	0.0108 (7)	−0.0060 (6)
O3	0.0281 (8)	0.0195 (7)	0.0340 (8)	−0.0028 (6)	−0.0025 (6)	−0.0016 (6)
O4	0.0271 (8)	0.0254 (8)	0.0696 (12)	−0.0013 (6)	−0.0025 (8)	0.0090 (8)
O5	0.0378 (9)	0.0210 (7)	0.0246 (8)	0.0022 (6)	0.0043 (6)	−0.0016 (6)
O6	0.0404 (9)	0.0219 (7)	0.0326 (9)	−0.0061 (6)	0.0069 (7)	0.0053 (7)
O7	0.0277 (8)	0.0197 (7)	0.0324 (8)	−0.0024 (5)	0.0017 (6)	−0.0075 (6)
O8	0.0297 (8)	0.0319 (8)	0.0341 (8)	−0.0020 (6)	0.0042 (6)	−0.0092 (7)
C1	0.0215 (10)	0.0228 (10)	0.0244 (10)	−0.0022 (7)	−0.0056 (8)	−0.0006 (8)
C2	0.0242 (10)	0.0206 (10)	0.0293 (11)	−0.0007 (8)	−0.0001 (8)	−0.0058 (8)
C3	0.0219 (10)	0.0246 (10)	0.0228 (10)	−0.0006 (7)	−0.0009 (8)	−0.0059 (8)
C4	0.0177 (9)	0.0252 (10)	0.0191 (9)	−0.0012 (7)	−0.0009 (7)	−0.0039 (8)
C5	0.0203 (10)	0.0226 (10)	0.0230 (10)	−0.0019 (7)	−0.0030 (8)	−0.0004 (8)
C6	0.0216 (10)	0.0196 (9)	0.0273 (10)	−0.0002 (7)	−0.0030 (8)	−0.0065 (8)
C7	0.0208 (10)	0.0254 (10)	0.0203 (9)	0.0005 (7)	−0.0030 (8)	−0.0051 (8)
C8	0.0176 (9)	0.0230 (9)	0.0226 (10)	−0.0013 (7)	−0.0049 (7)	−0.0010 (8)
C9	0.0172 (9)	0.0216 (9)	0.0195 (9)	−0.0022 (7)	−0.0020 (7)	−0.0030 (7)
C10	0.0282 (11)	0.0193 (9)	0.0245 (10)	0.0001 (8)	0.0006 (8)	−0.0030 (8)
C11	0.0224 (10)	0.0199 (9)	0.0273 (10)	0.0001 (7)	−0.0023 (8)	−0.0020 (8)
C12	0.0176 (9)	0.0231 (9)	0.0238 (10)	−0.0003 (7)	−0.0011 (8)	−0.0007 (8)
C13	0.0204 (10)	0.0239 (10)	0.0265 (10)	−0.0007 (7)	−0.0003 (8)	−0.0045 (8)
C14	0.0225 (10)	0.0286 (10)	0.0223 (10)	−0.0003 (8)	0.0027 (8)	−0.0044 (8)
C15	0.0215 (10)	0.0275 (10)	0.0253 (10)	−0.0043 (8)	−0.0009 (8)	0.0029 (8)
C16	0.0220 (10)	0.0190 (9)	0.0290 (10)	−0.0026 (7)	−0.0040 (8)	−0.0015 (8)
C17	0.0200 (10)	0.0247 (10)	0.0260 (10)	0.0010 (7)	−0.0014 (8)	−0.0049 (8)
C18	0.0320 (12)	0.0230 (10)	0.0216 (10)	0.0009 (8)	0.0033 (8)	0.0048 (8)
C19	0.0467 (14)	0.0242 (11)	0.0276 (11)	0.0005 (9)	0.0020 (10)	−0.0005 (9)
C20	0.0207 (10)	0.0185 (9)	0.0238 (10)	−0.0008 (7)	−0.0029 (8)	−0.0056 (8)
C21	0.0252 (10)	0.0179 (9)	0.0286 (10)	−0.0026 (7)	−0.0034 (8)	−0.0019 (8)
C22	0.0246 (10)	0.0197 (9)	0.0252 (10)	−0.0033 (7)	−0.0020 (8)	−0.0017 (8)
C23	0.0190 (9)	0.0247 (10)	0.0211 (9)	0.0003 (7)	−0.0010 (7)	−0.0052 (8)
C24	0.0207 (10)	0.0213 (9)	0.0242 (10)	0.0015 (7)	−0.0041 (8)	−0.0080 (8)
C25	0.0219 (10)	0.0146 (8)	0.0290 (10)	−0.0008 (7)	−0.0029 (8)	−0.0035 (8)
C26	0.0218 (10)	0.0196 (9)	0.0235 (10)	0.0006 (7)	−0.0045 (8)	−0.0024 (8)
C27	0.0176 (9)	0.0195 (9)	0.0238 (10)	0.0008 (7)	−0.0052 (7)	−0.0059 (8)
C28	0.0185 (9)	0.0191 (9)	0.0229 (10)	−0.0007 (7)	−0.0051 (7)	−0.0048 (7)
C29	0.0228 (10)	0.0155 (9)	0.0277 (10)	−0.0013 (7)	−0.0016 (8)	−0.0049 (8)
C30	0.0232 (10)	0.0159 (9)	0.0267 (10)	−0.0028 (7)	−0.0021 (8)	−0.0031 (8)
C31	0.0177 (9)	0.0183 (9)	0.0250 (10)	−0.0019 (7)	−0.0030 (7)	−0.0015 (8)
C32	0.0206 (10)	0.0231 (10)	0.0261 (10)	−0.0032 (7)	−0.0012 (8)	0.0042 (8)
C33	0.0253 (11)	0.0363 (11)	0.0214 (10)	−0.0038 (8)	0.0034 (8)	0.0040 (9)
C34	0.0262 (11)	0.0360 (11)	0.0206 (10)	−0.0022 (8)	0.0019 (8)	−0.0071 (9)
C35	0.0233 (10)	0.0191 (9)	0.0261 (10)	−0.0019 (7)	−0.0006 (8)	−0.0033 (8)
C36	0.0242 (10)	0.0191 (9)	0.0206 (9)	−0.0030 (7)	0.0016 (8)	0.0000 (7)
C37	0.0299 (11)	0.0274 (10)	0.0203 (10)	−0.0044 (8)	0.0017 (8)	−0.0066 (8)
C38	0.0366 (12)	0.0263 (11)	0.0342 (12)	−0.0066 (9)	0.0011 (9)	−0.0077 (9)

### Geometric parameters (Å, °)

**Table:**

Cl1—C6	1.7334 (19)	C13—C14	1.386 (3)
Cl2—C4	1.744 (2)	C14—C15	1.390 (3)
Cl3—C25	1.7334 (19)	C14—H14A	0.9500
Cl4—C23	1.745 (2)	C15—C16	1.386 (3)
N1—C1	1.339 (2)	C15—H15A	0.9500
N1—C8	1.364 (2)	C16—C17	1.373 (3)
N2—C20	1.337 (2)	C17—H17A	0.9500
N2—C27	1.359 (2)	C18—C19	1.491 (3)
O1—C7	1.356 (2)	C19—H19A	0.9800
O1—H1	0.78 (3)	C19—H19B	0.9800
O2—C13	1.359 (2)	C19—H19C	0.9800
O2—H2	0.88 (3)	C20—C21	1.413 (3)
O3—C18	1.349 (2)	C20—C29	1.465 (3)
O3—C16	1.415 (2)	C21—C22	1.366 (3)
O4—C18	1.203 (2)	C21—H21A	0.9500
O5—C26	1.345 (2)	C22—C28	1.414 (3)
O5—H5	0.77 (3)	C22—H22A	0.9500
O6—C32	1.375 (2)	C23—C24	1.362 (3)
O6—H6B	0.844 (10)	C23—C28	1.418 (3)
O6—H6A	0.829 (19)	C24—C25	1.406 (3)
O7—C37	1.354 (2)	C24—H24A	0.9500
O7—C35	1.416 (2)	C25—C26	1.374 (3)
O8—C37	1.208 (2)	C26—C27	1.428 (3)
C1—C2	1.413 (3)	C27—C28	1.415 (3)
C1—C10	1.466 (3)	C29—C30	1.328 (3)
C2—C3	1.362 (3)	C29—H29A	0.9500
C2—H2A	0.9500	C30—C31	1.461 (3)
C3—C9	1.418 (3)	C30—H30A	0.9500
C3—H3A	0.9500	C31—C32	1.405 (3)
C4—C5	1.367 (3)	C31—C36	1.408 (2)
C4—C9	1.413 (3)	C32—C33	1.389 (3)
C5—C6	1.405 (3)	C33—C34	1.373 (3)
C5—H5A	0.9500	C33—H33A	0.9500
C6—C7	1.366 (3)	C34—C35	1.384 (3)
C7—C8	1.426 (3)	C34—H34A	0.9500
C8—C9	1.414 (3)	C35—C36	1.373 (3)
C10—C11	1.330 (3)	C36—H36A	0.9500
C10—H10A	0.9500	C37—C38	1.493 (3)
C11—C12	1.463 (3)	C38—H38A	0.9800
C11—H11A	0.9500	C38—H38B	0.9800
C12—C17	1.402 (3)	C38—H38C	0.9800
C12—C13	1.409 (3)		
			
C1—N1—C8	117.82 (16)	C18—C19—H19B	109.5
C20—N2—C27	118.02 (16)	H19A—C19—H19B	109.5
C7—O1—H1	103 (2)	C18—C19—H19C	109.5
C13—O2—H2	112.3 (18)	H19A—C19—H19C	109.5
C18—O3—C16	117.18 (15)	H19B—C19—H19C	109.5
C26—O5—H5	104 (2)	N2—C20—C21	121.72 (17)
C32—O6—H6B	109.3 (12)	N2—C20—C29	118.39 (17)
C32—O6—H6A	110 (4)	C21—C20—C29	119.87 (17)
H6B—O6—H6A	118 (4)	C22—C21—C20	120.43 (17)
C37—O7—C35	117.40 (15)	C22—C21—H21A	119.8
N1—C1—C2	121.63 (18)	C20—C21—H21A	119.8
N1—C1—C10	118.84 (17)	C21—C22—C28	119.35 (18)
C2—C1—C10	119.52 (17)	C21—C22—H22A	120.3
C3—C2—C1	120.62 (18)	C28—C22—H22A	120.3
C3—C2—H2A	119.7	C24—C23—C28	121.65 (18)
C1—C2—H2A	119.7	C24—C23—Cl4	118.94 (15)
C2—C3—C9	119.60 (18)	C28—C23—Cl4	119.40 (14)
C2—C3—H3A	120.2	C23—C24—C25	119.58 (17)
C9—C3—H3A	120.2	C23—C24—H24A	120.2
C5—C4—C9	121.84 (18)	C25—C24—H24A	120.2
C5—C4—Cl2	119.16 (15)	C26—C25—C24	121.94 (17)
C9—C4—Cl2	119.00 (14)	C26—C25—Cl3	119.34 (15)
C4—C5—C6	119.44 (18)	C24—C25—Cl3	118.70 (14)
C4—C5—H5A	120.3	O5—C26—C25	121.47 (17)
C6—C5—H5A	120.3	O5—C26—C27	120.29 (17)
C7—C6—C5	121.55 (17)	C25—C26—C27	118.25 (18)
C7—C6—Cl1	119.40 (15)	N2—C27—C28	123.85 (17)
C5—C6—Cl1	119.05 (15)	N2—C27—C26	115.49 (17)
O1—C7—C6	122.30 (17)	C28—C27—C26	120.66 (17)
O1—C7—C8	118.63 (18)	C22—C28—C27	116.62 (17)
C6—C7—C8	119.06 (18)	C22—C28—C23	125.54 (18)
N1—C8—C9	124.12 (17)	C27—C28—C23	117.83 (17)
N1—C8—C7	115.63 (17)	C30—C29—C20	124.17 (17)
C9—C8—C7	120.25 (18)	C30—C29—H29A	117.9
C4—C9—C8	117.80 (17)	C20—C29—H29A	117.9
C4—C9—C3	126.01 (18)	C29—C30—C31	126.50 (17)
C8—C9—C3	116.19 (18)	C29—C30—H30A	116.7
C11—C10—C1	123.99 (18)	C31—C30—H30A	116.7
C11—C10—H10A	118.0	C32—C31—C36	117.29 (17)
C1—C10—H10A	118.0	C32—C31—C30	120.27 (16)
C10—C11—C12	126.92 (18)	C36—C31—C30	122.42 (17)
C10—C11—H11A	116.5	O6—C32—C33	118.40 (17)
C12—C11—H11A	116.5	O6—C32—C31	120.19 (18)
C17—C12—C13	117.82 (18)	C33—C32—C31	121.41 (17)
C17—C12—C11	123.98 (18)	C34—C33—C32	120.34 (18)
C13—C12—C11	118.20 (17)	C34—C33—H33A	119.8
O2—C13—C14	121.97 (18)	C32—C33—H33A	119.8
O2—C13—C12	116.57 (18)	C33—C34—C35	118.78 (19)
C14—C13—C12	121.45 (17)	C33—C34—H34A	120.6
C13—C14—C15	119.90 (18)	C35—C34—H34A	120.6
C13—C14—H14A	120.0	C36—C35—C34	122.11 (18)
C15—C14—H14A	120.0	C36—C35—O7	121.07 (17)
C16—C15—C14	118.57 (18)	C34—C35—O7	116.78 (17)
C16—C15—H15A	120.7	C35—C36—C31	120.05 (18)
C14—C15—H15A	120.7	C35—C36—H36A	120.0
C17—C16—C15	122.35 (18)	C31—C36—H36A	120.0
C17—C16—O3	119.97 (17)	O8—C37—O7	123.06 (18)
C15—C16—O3	117.62 (17)	O8—C37—C38	124.83 (19)
C16—C17—C12	119.90 (18)	O7—C37—C38	112.10 (17)
C16—C17—H17A	120.1	C37—C38—H38A	109.5
C12—C17—H17A	120.1	C37—C38—H38B	109.5
O4—C18—O3	122.59 (18)	H38A—C38—H38B	109.5
O4—C18—C19	126.15 (19)	C37—C38—H38C	109.5
O3—C18—C19	111.19 (18)	H38A—C38—H38C	109.5
C18—C19—H19A	109.5	H38B—C38—H38C	109.5
			
C8—N1—C1—C2	−1.0 (3)	C27—N2—C20—C21	−1.0 (3)
C8—N1—C1—C10	178.83 (16)	C27—N2—C20—C29	177.25 (16)
N1—C1—C2—C3	1.4 (3)	N2—C20—C21—C22	0.3 (3)
C10—C1—C2—C3	−178.34 (18)	C29—C20—C21—C22	−177.92 (17)
C1—C2—C3—C9	−0.5 (3)	C20—C21—C22—C28	0.2 (3)
C9—C4—C5—C6	−2.0 (3)	C28—C23—C24—C25	−1.4 (3)
Cl2—C4—C5—C6	178.09 (14)	Cl4—C23—C24—C25	177.52 (14)
C4—C5—C6—C7	0.8 (3)	C23—C24—C25—C26	−1.2 (3)
C4—C5—C6—Cl1	−179.90 (14)	C23—C24—C25—Cl3	179.99 (14)
C5—C6—C7—O1	−179.23 (17)	C24—C25—C26—O5	−177.55 (17)
Cl1—C6—C7—O1	1.5 (3)	Cl3—C25—C26—O5	1.3 (3)
C5—C6—C7—C8	1.3 (3)	C24—C25—C26—C27	3.0 (3)
Cl1—C6—C7—C8	−178.01 (14)	Cl3—C25—C26—C27	−178.21 (14)
C1—N1—C8—C9	−0.4 (3)	C20—N2—C27—C28	1.2 (3)
C1—N1—C8—C7	179.42 (17)	C20—N2—C27—C26	−178.79 (16)
O1—C7—C8—N1	−1.6 (3)	O5—C26—C27—N2	−1.7 (3)
C6—C7—C8—N1	177.91 (17)	C25—C26—C27—N2	177.76 (16)
O1—C7—C8—C9	178.25 (16)	O5—C26—C27—C28	178.26 (17)
C6—C7—C8—C9	−2.2 (3)	C25—C26—C27—C28	−2.3 (3)
C5—C4—C9—C8	1.0 (3)	C21—C22—C28—C27	0.0 (3)
Cl2—C4—C9—C8	−179.07 (13)	C21—C22—C28—C23	179.39 (18)
C5—C4—C9—C3	−179.37 (18)	N2—C27—C28—C22	−0.7 (3)
Cl2—C4—C9—C3	0.6 (3)	C26—C27—C28—C22	179.30 (16)
N1—C8—C9—C4	−179.03 (16)	N2—C27—C28—C23	179.81 (17)
C7—C8—C9—C4	1.1 (3)	C26—C27—C28—C23	−0.2 (3)
N1—C8—C9—C3	1.3 (3)	C24—C23—C28—C22	−177.38 (18)
C7—C8—C9—C3	−178.55 (17)	Cl4—C23—C28—C22	3.7 (3)
C2—C3—C9—C4	179.58 (18)	C24—C23—C28—C27	2.0 (3)
C2—C3—C9—C8	−0.8 (3)	Cl4—C23—C28—C27	−176.89 (13)
N1—C1—C10—C11	0.2 (3)	N2—C20—C29—C30	0.4 (3)
C2—C1—C10—C11	−179.98 (18)	C21—C20—C29—C30	178.71 (18)
C1—C10—C11—C12	179.48 (18)	C20—C29—C30—C31	−177.19 (17)
C10—C11—C12—C17	5.4 (3)	C29—C30—C31—C32	−175.38 (19)
C10—C11—C12—C13	−174.54 (19)	C29—C30—C31—C36	6.1 (3)
C17—C12—C13—O2	−179.68 (17)	C36—C31—C32—O6	178.42 (17)
C11—C12—C13—O2	0.3 (3)	C30—C31—C32—O6	−0.2 (3)
C17—C12—C13—C14	−0.7 (3)	C36—C31—C32—C33	−0.8 (3)
C11—C12—C13—C14	179.21 (17)	C30—C31—C32—C33	−179.41 (18)
O2—C13—C14—C15	180.00 (18)	O6—C32—C33—C34	−177.64 (18)
C12—C13—C14—C15	1.1 (3)	C31—C32—C33—C34	1.6 (3)
C13—C14—C15—C16	−0.6 (3)	C32—C33—C34—C35	−0.9 (3)
C14—C15—C16—C17	−0.3 (3)	C33—C34—C35—C36	−0.5 (3)
C14—C15—C16—O3	177.00 (16)	C33—C34—C35—O7	−178.19 (17)
C18—O3—C16—C17	−79.5 (2)	C37—O7—C35—C36	76.0 (2)
C18—O3—C16—C15	103.1 (2)	C37—O7—C35—C34	−106.3 (2)
C15—C16—C17—C12	0.6 (3)	C34—C35—C36—C31	1.3 (3)
O3—C16—C17—C12	−176.57 (16)	O7—C35—C36—C31	178.87 (16)
C13—C12—C17—C16	−0.1 (3)	C32—C31—C36—C35	−0.6 (3)
C11—C12—C17—C16	179.92 (18)	C30—C31—C36—C35	177.95 (17)
C16—O3—C18—O4	−0.1 (3)	C35—O7—C37—O8	−5.6 (3)
C16—O3—C18—C19	−177.25 (17)	C35—O7—C37—C38	173.12 (16)

### Hydrogen-bond geometry (Å, °)

**Table:**

*D*—H···*A*	*D*—H	H···*A*	*D*···*A*	*D*—H···*A*
O1—H1···N1	0.78 (3)	2.14 (3)	2.667 (2)	125 (3)
O2—H2···O8	0.88 (3)	1.84 (3)	2.675 (2)	159 (3)
O5—H5···N2	0.77 (3)	2.20 (3)	2.692 (2)	122 (3)
O5—H5···O4^i^	0.77 (3)	2.38 (3)	2.990 (2)	138 (3)
O6—H6A···O6^ii^	0.83 (2)	2.12 (3)	2.892 (3)	156 (5)
O6—H6B···O4^i^	0.84 (1)	2.44 (1)	3.130 (3)	139 (2)

Symmetry codes: (i) *x*+1, *y*, *z*; (ii) −*x*+2, −*y*+1, −*z*.
